# Sugarsquare, a Web-Based Patient Portal for Parents of a Child With Type 1 Diabetes: Multicenter Randomized Controlled Feasibility Trial

**DOI:** 10.2196/jmir.6639

**Published:** 2017-08-22

**Authors:** Emiel Boogerd, Nienke M Maas-Van Schaaijk, Theo C Sas, Agnes Clement-de Boers, Mischa Smallenbroek, Roos Nuboer, Cees Noordam, Chris M Verhaak

**Affiliations:** ^1^ Department of Medical Psychology Radboud University Medical Center Nijmegen Netherlands; ^2^ Children's Diabetes Center Nijmegen Nijmegen Netherlands; ^3^ Department of Pediatrics Albert Schweitzer Hospital Dordrecht Netherlands; ^4^ Department of Pediatrics Juliana Childrens' Hospital The Hague Netherlands; ^5^ Department of Pediatrics Medical Center Leeuwarden Leeuwarden Netherlands; ^6^ Department of Pediatrics Meander Medical Center Amersfoort Netherlands; ^7^ Department of Pediatrics Radboud University Medical Center Nijmegen Netherlands

**Keywords:** diabetes mellitus, type 1, parenting, health communication, peer group, telemedicine, Internet

## Abstract

**Background:**

Raising a child with type 1 diabetes (T1D) means combining the demands of the disease management with everyday parenting, which is associated with increased levels of distress. A Web-based patient portal, Sugarsquare, was developed to support parents, by providing online parent-professional communication, online peer support and online disease information.

**Objective:**

The first aim of this study was to assess the feasibility of conducting a multicenter, randomized controlled trial in Dutch parents of a child with T1D. The second aim was to assess the feasibility of implementing Sugarsquare in clinical practice.

**Methods:**

The parents of 105 children (N=105) with T1D below the age of 13 participated in a 6-month multicenter randomized controlled feasibility trial. They were randomly assigned to an experimental (n=54, usual care and Sugarsquare) or a control group (n=51, usual care). Attrition rates and user statistics were gathered to evaluate feasibility of the trial and implementation. To determine potential efficacy, the parenting stress index (PSI-SF) was assessed at baseline (T0) and after 6 months (T1).

**Results:**

Of a potential population of parents of 445 children, 189 were willing to participate (enrollment refusal=57.5%, n=256), 142 filled in the baseline questionnaire (baseline attrition rate=25%, n=47), and 105 also filled in the questionnaire at T1 (post randomization attrition rate during follow-up=26%, n=32). As such, 24% of the potential population participated. Analysis in the experimental group (n=54) revealed a total of 32 (59%) unique users, divided into 12 (38%) frequent users, 9 (28%) incidental users, and 11 (34%) low-frequent users. Of the total of 44 professionals, 34 (77%) logged in, and 32 (73%) logged in repeatedly. Analysis of the user statistics in the experimental group further showed high practicability and integration in all users, moderate acceptability and demand in parents, and high acceptability and demand in health care professionals. Baseline parenting stress index scores were related to the parents’ frequency of logging on (*ρ*=.282, *P*=.03) and page-views (*ρ*=.304, *P*=.01). No significant differences in change in parenting stress between experimental and control group were found (*F*_3,101_=.49, *P*=.49).

**Conclusions:**

The trial can be considered feasible, considering the average enrollment refusal rate, baseline attrition rate and postrandomization attrition rate, compared to other eHealth studies, although lower than hypothesized. Implementing Sugarsquare in clinical practice was partly feasible, given moderate demand and acceptability in parent users and lack of potential efficacy. Parents who reported higher levels of parenting stress used Sugarsquare more often than other parents, although Sugarsquare did not reduce parenting stress. These results indicate that Web-based interventions are a suitable way of providing parents of children with T1D with additional support. Future studies should determine how Sugarsquare could reduce parenting stress, for instance by adding targeted interventions. Factors potentially contributing to successful implementation are suggested.

**Trial Registration:**

Nederlands Trial Register Number: NTR3643; http://www.trialregister.nl/trialreg/admin/rctview.asp?TC=3643 (Archived by WebCite at http://www.webcitation.org/6qihOVCi6)

## Introduction

### Background

Type 1 diabetes (T1D) is a chronic metabolic disorder with a complex daily treatment regime, requiring patients to carry out a variety of health-related self-care behaviors, such as monitoring blood glucose levels, administering insulin, adhering to a diet, and exercising. In case of young children, parents are responsible for ensuring that these disease management tasks are performed. Having to combine these complex self-management tasks with regular parenting tasks in everyday life can have a profound impact on parents [1-10], indicated by elevated levels of stress and depressive symptoms in parents of a child with T1D [3,7,9,11], especially in those with young children and with children with a more recent diagnosis [2-7,12,13]. Family and parental functioning are related to well-being, self-care skills, and glycemic control in children, which makes it important that diabetes teams are aware of the impact of the disease and its treatment on parents [1,6,14-19]. Studies show that parents need easy access to their diabetes care team [8,20,21], local peer support [22-26], and tailored information about the disease and its management provided by their own diabetes team [8,27-30]. This positively affects their quality of life [8,23,26] and helps them adequately cope with the disease.

New technologies such as the Internet can help diabetes teams in delivering these aspects [8,25,26,29,31-40]. Despite the great potential of the Internet and parents’ positive attitude toward using Internet in care, there has been little research into the efficacy and feasibility of Internet interventions for the parents of chronically ill children, especially interventions that combine multiple aspects of care [38,39,41]. This is unfortunate, considering that chronically ill patients and their parents can benefit from using the Internet, because it facilitates the exchange of knowledge and information between patients and health care professionals.

There are several challenges, when it comes to implementing and testing an Internet intervention in a clinical research context. eHealth studies are specifically subject to low retention rates (evaluation dropout), which are often the result of study-specific factors and low adherence rates (nonintervention usage) that are mostly intervention specific. These rates can lead to a loss of participants and thus to lack of statistical power [34,42-46]. Achieving successful recruitment is particularly problematic when multiple practices are involved, as practices often differ at an organizational level and local recruiters often have limited resources for recruitment [47,48].

### Randomized Controlled Trial

To gain knowledge about the feasibility of conducting a randomized controlled trial (RCT) and implementing an Internet intervention in usual care for parents of a child with T1D, we developed a Web-based patient portal, called Sugarsquare [40]. Sugarsquare was specifically developed according to parents’ needs and preferences [8,31] and is hypothesized to enable diabetes teams to improve their accessibility, facilitate local peer support, and provide tailored information [31]. An explorative, multicenter study was conducted to answer the following research questions:

1. Is conducting an RCT concerning Sugarsquare feasible in a population of parents of a child with T1D in terms of:
  - potential participants: what is the number of eligible parents?
  - enrollment refusal rate: what is the proportion of parents who refuse participation?
  - baseline attrition rate: what is the proportion of parents who drop out before baseline?
  - follow-up attrition rate: what is the proportion of parents who drop out during the trial?
2. Is implementation of Sugarsquare in daily clinical practice feasible in a population of parents of a child with T1D in terms of:
  - practicability: are recipients able to use Sugarsquare?
  - acceptability: do recipients use Sugarsquare?
  - demand: do recipients continue to use Sugarsquare?
  - integration: is Sugarsquare consistent with international guidelines for pediatric diabetes care?
  - potential efficacy: is usage associated with change in parenting stress?

## Methods

### Design and Setting of the Study

The participants for this study were recruited from 7 medical centers in the Netherlands, with a potential of 445 parents, from May 2012 to January 2013. Eligible participants were the parents of a child with T1D (one parent per child) younger than 13 years of age, had access to the Internet at home, and were able to comprehend the Dutch language. The children had to be treated in one of the participating centers during the entire course of the study. Participants were randomly assigned to one of two conditions: (1) an intervention condition and (2) a usual care control condition. Participants in the intervention condition had access to the intervention for 6 months in addition to care as usual. Participants in the control group received care as usual during that period. An extensive report of the offline recruitment of participants, the randomization and the procedure of the data collection is described in the Sugarsquare study protocol [31]. The study described in this study was part of a larger project [31], of which all procedures were approved by the Ethics Committees of Human Experimentation of the Radboud University Medical Center and the participating hospitals and are in accordance with the Declaration of Helsinki.

### Intervention

The final version of Sugarsquare consists of a Web-based patient portal providing online parent-professional communication, peer support, and disease information. Sugarsquare was developed at parents’ explicit request and is based on a previous comparable intervention for adolescents with T1D [8,31,40]. Seven focus group interviews with parents [8,31] and a questionnaire for health care professionals affiliated to the cooperating centers were used to tailor the intervention to the preferences of both parents and health care professionals. In a series of pilots, involving parents and professionals participated, the intervention was further fine-tuned and facilitators and barriers were identified. The test phase ended when bugs were repaired and both parents and professionals felt the intervention was ready for use. In accordance with parents’ preferences, the intervention was organized locally, so that each center for diabetes care has its own secured portal, which is only accessible to health care professionals of that particular center and to the parents of the children treated at that clinic. Sugarsquare is accessible through the Internet and has the following two main sections.

#### Section I: General

The first section provides online peer support and disease information and is accessible to all users (parents and health care professionals). Peer support is facilitated through a chat application, a forum application, and a blog application. Disease information is provided by means of downloadable documents and Web links.

#### Section II: Personal

The second section is specific to individual patients and can only be accessed by the parents of that particular patient and their diabetes team. The section contains an overview of treatment goals and an application for easily accessible private contact between parents and health care professionals. This application is only used for nonurgent matters.

The intervention has been described in the study protocol [31]. In the final version of Sugarsquare, disease information is incorporated in Section I, instead of Section II as described in the study protocol. Sugarsquare is secured by means of a 2-factor authentication, requiring a username-password combination and a personalized SMS code in the login procedure. Health care professionals of the local diabetes teams were appointed as coordinators for the local recruitment of participants and the local implementation of Sugarsquare. Screenshots of Sugarsquare for parents are displayed in Multimedia Appendix 1 [49].

### Care as Usual

All children received care as usual, according to International Guidelines for Pediatric Diabetes Care [18,50], provided by a multidisciplinary team of pediatric diabetologists, diabetes nurse practitioners, dietitians, and psychologists. Parents and children were invited to visit the outpatient center for consultations with the pediatric diabetologist and nurse practitioner 4 times a year. Dieticians and psychologists were available on request by parents, children, or physicians. The diabetes care team could be contacted during business hours by telephone and email. An emergency telephone number could be accessed outside office hours to guarantee continuous access to care. Children of participants in both conditions (experimental and control) received care as usual during the entire study period. As such, Sugarsquare was used in addition to care as usual. During the study period, the parents in the experimental group could contact the diabetes care team via the portal instead of by telephone or email in case of nonurgent matters. The telephone number for emergencies was maintained.

### Measures

Feasibility of the RCT was assessed in terms of the number of potential participants, the proportion of parents who refused participation, and the attrition rates. Demographic data of all the participants who were included in the final analyses were gathered at baseline.

For assessment of feasibility of the intervention, expressed in terms of practicability, acceptability, and demand [40,51], individual user data of all participants in the experimental group, such as frequency of logins and number of messages posted on the forum, were logged digitally. For feasibility in terms of integration, we assessed whether Sugarsquare was of added value for working according to International ISPAD (International Society for Pediatric and Adolescent Diabetes) and or IDF (International Diabetes Federation) and ADA (American Diabetes Association) Guidelines for Diabetes Care [18,50], by checking 9 key-elements for diabetes care, derived from these guidelines. For feasibility in terms of potential efficacy, parenting stress was assessed by means of the Dutch version of the parenting stress index-short form (PSI-SF) [52] on T0, T1, and T2. The reliability and criterion validity of the Dutch PSI-SF are shown to be good [52]. The PSI-SF consists of 25 items answered on a 6-point Likert scale, ranging from “totally agree” to “totally disagree.” An example of an item on the PSI-SF is “it is not always easy to accept my child the way he or she is.” The sum score on the PSI-SF can be categorized into normal, subclinical, and clinical based on standardized cutoff scores described in the manual [52]. Parenting stress was assessed at the start of the study (T0=baseline), at 6 months after the start of the study (T1), and at 12 months after the start of the study (T2=follow-up). Also, at the end of the study we asked the local Sugarsquare coordinators, who were health care professionals and part of the local diabetes teams, to evaluate the study and identify facilitators and limitations for the implementation.

Information about the child’s glycemic control (HbA1c) and the number of hospital admissions (lasting over 24 h) for keto-acidosis or severe hypoglycemia were used to explore the potential efficacy of the portal. These data were taken from the child’s medical files.

Questionnaires for demographics and parenting stress were administered by means of a Web-based, secured survey program, called Radquest, which generates a closed survey system. The registered participants received an email with a Web link to the survey, which was paired with a unique user id. All items had to be answered and participants were able to change the answers until the participant submitted the completed survey. The data generated from the survey were stored on a secured server.

Some participants preferred filling in a hardcopy questionnaire, which was sent to them by post. For an elaborate overview of all measures, see Table 1.

### Analyses

Demographic data were analyzed descriptively, and differences at baseline between the experimental group and the control group were assessed using an analysis of variance (ANOVA). For feasibility, user data were analyzed by means of descriptive statistics. To compare differences in change in parenting stress between the experimental group and the control group, an analysis of covariance (ANCOVA) was performed on T1 data, using T0 data as covariate and the condition (experimental vs control) as fixed factor. A sensitivity analysis was conducted by means of a multiple imputation analysis (based on HbA1c scores at T1) to account for missing data. To test robustness of the results, a conservative analysis based on a Last Observation Carried Forward (LOCF) imputation was performed. Associations between user data and parenting stress at baseline were explored using Spearman *ρ* for nonparametric correlation due to high skewness of user data and a univariate ANOVA. Data on T2 were regarded as follow-up and were not analyzed in this study.

### Power Calculation

We calculated that the data of 180 parents would be needed for the final analysis in order to reach a medium effect size (*d*=0.50), with a Cronbach alpha of .05 (two-tailed test) and a beta of .10 [31]. On the basis of recent literature, a declination rate of 25% (n=80) and a dropout rate of 25% (n=60) was hypothesized [31,34]. As such, we would need to approach 320 parents in order to reach a minimum of 240 parents at the start of the study to have data for 180 participants in the final analysis [31].

**Table 1:** Variables used in the Sugarsquare study.

Outcome			Measures
**Demographics**			
		Age and gender of the child	
		Age of onset and duration of diabetes	
		Pen or pump treatment	
		Age, gender, and educational level of the primary parent	
		Social economic status of the parents	
**Feasibility of the trial**			
	Potential population	Total population of parents, N (%)	
	Enrollment refusal	Participants who consented (total population of parents), n (%)	
	Baseline attrition	Participants who completed T0 (participants enrolled), n (%)	
	Postrandomization attrition (during follow-up)	Participants who completed T1 (randomized participants), n (%)	
**Feasibility of intervention**			
	Practicability (can they use it?)	Inventory of difficulties logging in and downtime (inaccessibility)	
	Acceptability (do they use it?)	Percentage of users who logged in at least once and used all applications	
	Demand or adherence (do they continue to use it?)	Percentage of users who logged in repeatedly	
	Integration (does it fit with the treatment?)	Evaluation of international guidelines for diabetes care (ISPAD or IDF and ADA) when using Sugarsquare	
	Potential efficacy (is usage associated with change in parenting stress?)	Parenting stress index-short form (PSI-SF [44])	
**Exploration of change in medical parameters**			
	Medical parameters	HbA1c	
		Hospitals admissions due to glycemic disruptions	

## Results

### Feasibility of the Randomized Controlled Trial: Enrollment and Dropout

**Figure 1:**
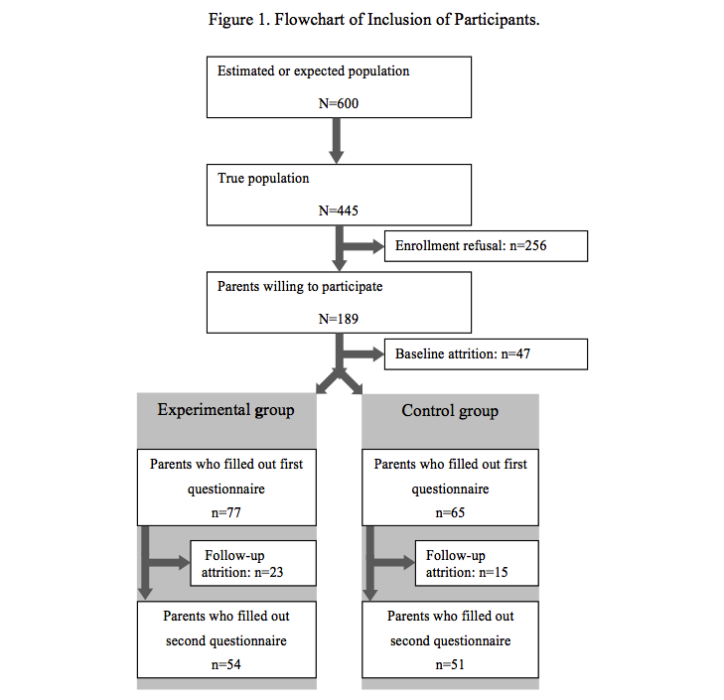
Flowchart of inclusion of participants.

All the parents of children with T1D, who were treated in 1 of the 7 cooperating centers for pediatric diabetes care, were invited by mail to participate in the study. The total population consisted of the parents of 445 children. A total of 189 parents of 189 children were willing to participate. The remaining 256 potential participants refused participation (enrollment refusal rate=57.5%). Frequently mentioned reasons for not participating were a lack of time, no interested in additional care and having to temporarily increase the focus on diabetes. A number of 142 parents filled in the baseline questionnaire. As such, 47 parents (baseline attrition rate=25%) dropped out before filling out the first questionnaire. Mentioned reasons for dropping out were a loss of interest and a lack of time. Subsequently, 105 parents also filled in the questionnaire at T1, meaning that 32 (postrandomization attrition rate during follow-up=26%) participants dropped out during the course of the study. Participants dropped out due to losing interest, a lack of time or because they changed from treatment center. As such, 23.6% (n=105) of the *potential population* successfully participated in the study (see also Figure 1).

### Demographics

The demographic statistics of the 105 participants are displayed in Table 2. A one-way, between-group ANOVA revealed no significant differences in any of the variables at baseline between the centers.

**Table 2:** Demographics and baseline scores of the participants.

Demographic variables			Experimental group		Control group	Total group
Parents (n)			54		51	105
Gender (male; female; filled in together)			49; 5		44; 5; 2	93; 10; 2
**Educational level**						
	Lower secondary education, n (%)	2 (4)		4 (8)		6 (6)
	Middle secondary education, n (%)	3 (5)		4 (8)		7 (7)
	Higher secondary education, n (%)	24 (44)		19 (37)		43 (41)
	Middle tertiary education, n (%)	9 (17)		2 (4)		11 (11)
	Higher tertiary education, n (%)	9 (17)		19 (37)		28 (27)
	Academia, n (%)	7 (13)		3 (6)		10 (10)
**Child**						
	Age in years, mean (SD^a^)	9,1 (2.9)		8,9 (2.5)		9 (2.7)
	Gender (female; male)	30; 24		27; 24		57; 48
	HbA1c in mmol/mol, mean (SD)	64 (13.77)		62 (7.77)		63 (10.62)
	HbAc in %, mean (SD)	7,98 (1.17)		7,86 (0.71)		7,92 (0.97)
**Insulin therapy**						
	Injections, n (%)	10 (19)		15 (29)		25 (24)
	Pump, n (%)	44 (82)		36 (71)		80 (76)

^a^SD: standard deviation.

### Feasibility of the Intervention

Data from the 54 participants in the experimental group and who therefore had access to Sugarsquare were used for the feasibility analysis and for the analysis relating user data and baseline scores on questionnaires. A proportion of 59% (n=32) of the parents who had access, used Sugarsquare during the trial (Table 3). Of the 32 unique parent users, 11 (34%) logged in repeatedly, at least once every 2 weeks and 9 (28%) logged in incidentally (3 times or more, but under once every 2 weeks), and 16 (41%) logged in once or twice during the study period. Table 3 also shows that 34 (77%) of 44 professionals who had received access at the start of the study, logged in and 32 (94%) logged in again. Thus, overall, 73% (n=32) of the professionals accessed Sugarsquare more than once. All users (parents and professionals) viewed all applications at least once when they logged in. The applications for forum (#page views=2838) and contact with the treatment team (#page views=2795) were viewed more often than the applications for information (#page views=415) and chat (#page views=683). Users reported no downtime, although 2 users reported that they sometimes could not access Sugarsquare, due to technical problems with the users’ telecom providers. Some parents (n=8) said that the two-step security procedure as a hassle. Sugarsquare attributed to provision of care according to all 9 key elements, derived from the Global IDF or ISPAD and ADA Guidelines for Diabetes care in Childhood and Adolescence (see also Multimedia Appendix 2) [18,50]. According to the Sugarsquare coordinators, there were 3 factors that limited implementation. These factors were the two-step login procedure, the lack of customized instructions for health care professionals and the randomization on individual level. The local Sugarsquare coordinators and the multidisciplinary approach of the team were suggested as 2 factors that positively affected implementation.

### Potential Efficacy

With regard to parenting stress, 82 (78%) parents (control and experimental condition) reported average or below average levels of parenting stress compared with Dutch healthy controls, 19 (18%) reported slightly elevated levels, and 4 (4%) reported very high levels of parenting stress (see also Table 4).

The analysis revealed no significant differences in change in parenting stress over time between the two groups (*F*_3,101_=.49, *P*=.49), or between centers (*F*_3,101_=.31, *P*=.91), and nor was there an interaction between groups and centers (*F*_3,101_=1.16, *P*=.34). Similar results were obtained in an ANCOVA (Table 5) without the factor center and a sensitivity analysis, conducted by means of a multiple imputation analysis. Since no change was found, a conservative analysis using LOCF was not conducted. We also found no significant differences in change over time in HbA1c levels between the experimental group and the control group (*F*_3,101_=.040, *P*=.84).

### Baseline Parenting Stress Levels and Portal Usage

The analysis revealed that parenting stress at baseline was significantly correlation with the frequency of logging in (*ρ*=.282, *P*=.03 Table 6) and the number of pages viewed (*ρ*=.304, *P*=.02). It seems that the greater stress parents experienced, the more parents logged in and the more pages they viewed.

**Table 3:** Sugarsquare usage during the first phase (6 months) of the study period.

User statistics		Parents		Professionals		Parents and professionals	
**Parents**							
	n (experimental group)		54		44		
	Unique visitors, n (%)		32 (59)		34 (77)		
**Log-ins**							
	High frequent users, n (%)		12 (38)		12 (35)		
	Moderate users, n (%)		9 (28)		20 (59)		
	Low frequent users, n (%)		11 (34)		2 (6)		
	#logins (n)		419		505		
	#logins, mean (SD^a^)		7,8 (13)		11,5 (16)		
**Page views**							
	#page views (n)		5690		8006		
	#mean page views, mean (SD)		105,4 (175)		182 (253)		
**Information**							
	#Documents visits (n)						415
	#Web links visits (n)						213
**Patient-professional contact**							
	#Questions visits (n)						2795
	#Questions input (n)						344
	#Treatment visits (n)						674
	#Treatment input (n)						29
**Peer support**							
	#Forum visits (n)						2838
	#Forum input (n)						147
	#Chat visits (n)						683
	#Chat input (n)						1653

^a^SD: standard deviation.

**Table 4:** Distribution of parenting stress index (PSI) scores for the total group.

PSI^a^-scores	n (%)
Normal stress scores	82 (78)
Elevated stress scores	19 (18)
High stress scores	4 (4)

^a^PSI: parenting stress index.

**Table 5:** Results of the analysis of covariance (ANCOVA) in parenting stress and HbA1c.

Efficacy variables	Experimental group		Control group		*F*
	T0	T1	T0	T1	
	Mean (SD^a^)	Mean (SD)	Mean (SD)	Mean (SD)	
PSI^b^	48.13 (19.46)	51.35 (22.32)	44.61 (17.60)	44.45 (17.89)	.49
HbA1c	63.74 (12.77)	63.06 (8.98)	62.41 (7.77)	62.54 (8.64)	.04

^a^SD: standard deviation.

^b^PSI: parenting stress index.

**Table 6:** Correlations of parenting stress at baseline and frequency of log-ins and page views.

Efficacy variables	#log-ins	#page views
Parenting stress (baseline)	*ρ*=.282, *P*=.03	*ρ*=.304, *P*=.02

## Discussion

### Principal Findings

This study investigated the feasibility of conducting a trial and implementing an Internet intervention in a population of parents of children with T1D, in daily clinical practice. It revealed that eHealth has the potential to create a platform for shared, daily disease management between professionals and parents. Sugarsquare seems to attract parents with relatively high stress levels. The participation rate and dropout rate in the RCT were average, compared with other trial studies and results indicated that conducting a trial concerning Sugarsquare was feasible. The implementation of Sugarsquare in clinical practice was partly feasible, given the high practicability in all users, moderate acceptability and demand in parent users, high acceptability and demand in professional users, high level of integration and lack of potential efficacy.

It is interesting to note that parents reporting higher levels of parenting stress were more likely to use Sugarsquare compared with parents reporting lower levels. This is consistent with a recent study by Balkhi and colleagues [26], who reported that parents with higher stress levels more frequently visited diabetes-related online forums than did parents with lower stress levels. As no association between HbA1c and usage was found, it is assumed that general parenting stress is associated with usage and not stress related to medical condition of the child. However, it is quite possible that the parents who did not use Sugarsquare might do so if they have a temporary need for additional support or information, for instance if their child becomes ill, at onset of puberty or if they are planning a trip abroad.

Our enrollment refusal rate (57.5%) and baseline attrition rate (25%) fell within the ranges described in the review by Karlson and Rapoff (2009), who found the refusal rates in eHealth studies to be ranging from 0% to 75% (mean 37%) and baseline attrition rates ranging from 0% to 35% (mean 4%) [53]. From this perspective, the rates in this study are reasonable. Still, we expected a lower enrollment refusal rate, since the intervention was requested by parents and fitted to their preferences by means of focus group interviews. It could be that the questionnaires, which had to be filled in by the parents on several occasions, discouraged potential participants [54]. It is also possible that, due to the research context, parents perceived this study as an externally driven project, which conflicted with their preference for a center-driven intervention [8] and might have negatively influenced their willingness to cooperate [55]. Our study was further confronted with an average postrandomization attrition rate during follow-up (26% vs 0-54%, mean 20% in Karlson and Rapoff) [34,53]. The eHealth studies are subject to low enrollment and high dropout rates. In order to resolve the issue of low enrollment, Lernmark and colleagues [56] suggested that clarity should be provided about what participants are expected to invest and about the potential added value of the study results for the individual participant, their clinic or care in general. Baxter and colleagues [57] suggested that interaction between researchers and participants is vital for keeping participants committed after they decide to participate.

During the study, possibilities to improve the trial and implementation were identified. First of all, customized instructions for when and how to use Sugarsquare, would have helped them fit Sugarsquare into their daily workflow and encourage parents to use Sugarsquare [58-60]. Also, Sugarsquare was used in a research context and randomization took place on an individual level. As such, only a part of the population in each center participated in this study. This meant that health care professionals had to work using two procedures simultaneously, making their work very complex and intensive and complicating the integration of Sugarsquare in their workflow of everyday [61,62]. The research context also had a negative effect on the amount of interaction on Sugarsquare, since only a relatively small population of parents had access to the platform. Implementation would have been more successful if randomization was conducted on center level, which would have meant that a center would have used Sugarsquare for its entire population or not at all.

Factors that might have contributed to the success of the trial and the implementation were also identified. The teams all appointed a team member dedicated to Sugarsquare, who coordinated local recruitment and implementation, and monitored Sugarsquare usage. This might have supported the teams in integrating the intervention in usual care, since studies in the past reported that this lead to increased awareness in the team for usage of innovative interventions [44,59,62]. Also, the multidisciplinary approach of the Diabetes teams in our study might have contributed to the implementation of Sugarsquare, since literature shows that members of multidisciplinary teams are used to working toward shared, organizational goals, which makes it easier to implement changes into their workflow [58,59].

Sugarsquare has a broad focus and consists of multiple, general, potentially feasible applications. These characteristics fit to the needs of the parents, as expressed in the focus groups [8]. However, because of this broad focus, it is difficult to establish which applications (information, peer contact, contact with staff) contributed to usage and to potential effect. As such, mechanisms of change could not be identified. Future studies could apply multiple study arms to adequately assess the value of single applications, which would increase the number of participants required. [63,64]. Another way of identifying potential working mechanisms and the value of single applications would be to collect qualitative data. This is expected to provide more insight into both and future researchers should consider collecting qualitative data in their study. In this study, we used a generic questionnaire to assess parenting stress, considering its broad use in pediatrics and the lack of a diabetes-specific one. Although generic parenting stress measures can be helpful for assessing stressors and distress, they might not be sensitive to issues specific to the parents of children with an illness or specific disease-related issues and, as such, failed to properly assess potential change in those domains [65]. Future studies could consider using an instrument designed for parents of a child with T1D or, in case this is lacking, an instrument for parents of pediatric patients, such as the Pediatric Inventory for Parents (PIP) or the recently validated pediatric parenting stress index (PPSI). The direct effect of the small sample size in this study is expected to be limited, since the sensitivity analyses did not show different outcomes compared with the completer analysis. However, indirectly, the limited number of participants in the local centers may have decreased the interaction on the local Sugarsquares and, with that, generalizability of the results. Future studies can avoid this by using randomization on center level.

Sugarsquare can be considered as a promising tool for diabetes teams, virtually extending their diabetes center. It contributes to usual care, because it offers parents and professionals a secured, Web-based platform for parent-health care professional communication, moderated peer support, and tailored disease information. In addition, it especially attracts parents who experience higher parenting stress levels. Given the complications that arose when Sugarsquare was used together with conventional communication tools, it is recommended that Sugarsquare be used as the sole medium for regular communication between parents and diabetes team. Appointing a dedicated Sugarsquare manager and using adequate instructions for the involved professionals are also hypothesized to contribute to the integration of Sugarsquare in care as usual. In order to increase usage by parent users and to improve their acceptance of Sugarsquare in daily care, diabetes teams could continuously add new content to Sugarsquare. This is expected to keep Sugarsquare interesting and to invite parent users to post information as well. It is also important that all team members post information, which shows parent users that Sugarsquare is accepted by the whole team. This might lower the threshold for parent users to use and accept Sugarsquare. This has been found to be workable in 9 centers for diabetes care in the Netherlands, which have implemented Sugarsquare in usual care.

In a recent study on the implementation of an eHealth intervention regarding online assessment of quality of life, it was noticed that successful implementation is affected by many factors acting on different aspects of implementing an intervention [66]. In general, they distinguish between factors on the level of the existing IT-structures (eg, usability, compatibility), organization (eg, support, expectations of management for usage), and the intervention itself (eg, easy to use, technical problems). As attrition rates as well as limited implementation are general challenges in eHealth, future studies should pay more attention to these factors. Another issue in the field of eHealth is that the financial costs of maintenance of interventions have yet to be included in systems for health care costs. The main problem that arises from this issue is the high number of interventions that are not implemented after a trail.

When starting an intervention study, we advise researchers to start with a single center trial for exploration of feasibility and potential efficacy. When feasibility and potential efficacy are demonstrated, a multicenter implementation could be conducted, potentially combined with assessment of efficacy using a historic design.

### Conclusions

This study concerned a generic intervention, based on parents’ preferences and needs, serving different aims, especially regarding shared disease management between parents and professionals. Our next step is to further develop the potential of Sugarsquare to serve as a platform for provision of more mechanism-focused interventions, targeted to reduce parenting stress, for instance, by providing online information or online cognitive behavior therapy. More generally, eHealth has possibilities to support monitoring of physical and psychosocial well-being, facilitate peer contact, interaction between patients and health care professionals and exchange of data. Sugarsquare can serve as central portal through which these applications or interventions can be accessed.

## Appendix

Multimedia Appendix 1 Screenshots of Sugarsquare for parents.

Multimedia Appendix 2 Check of the International Guideline and Standards for Diabetes Care.

Multimedia Appendix 3 CONSORT-EHEALTH checklist (v1.6.1).

## Abbreviations

ANCOVA: analysis of covariance
ANOVA: analysis of variance
LOCF: last observation carried factor
PSI-SF: parenting stress index-short form
T1D: type 1 diabetes

## References

[1] Helgeson VS, Becker D, Escobar O, Siminerio L (2012). Families with children with diabetes: implications of parent stress for parent and child health. J Pediatr Psychol.

[2] Hilliard ME, Monaghan M, Cogen FR, Streisand R (2011). Parent stress and child behaviour among young children with type 1 diabetes. Child Care Health Dev.

[3] Hullmann SE, Wolfe-Christensen C, Ryan JL, Fedele DA, Rambo PL, Chaney JM, Mullins LL (2010). Parental overprotection, perceived child vulnerability, and parenting stress: a cross-illness comparison. J Clin Psychol Med Settings.

[4] Mitchell SJ, Hilliard ME, Mednick L, Henderson C, Cogen FR, Streisand R (2009). Stress among fathers of young children with type 1 diabetes. Fam Syst Health.

[5] Powers SW, Byars KC, Mitchell MJ, Patton SR, Standiford DA, Dolan LM (2002). Parent report of mealtime behavior and parenting stress in young children with type 1 diabetes and in healthy control subjects. Diabetes Care.

[6] Streisand R, Mackey ER, Elliot BM, Mednick L, Slaughter IM, Turek J, Austin A (2008). Parental anxiety and depression associated with caring for a child newly diagnosed with type 1 diabetes: opportunities for education and counseling. Patient Educ Couns.

[7] Whittemore R, Jaser S, Chao A, Jang M, Grey M (2012). Psychological experience of parents of children with type 1 diabetes: a systematic mixed-studies review. Diabetes Educ.

[8] Boogerd EA, Maas-van Schaaijk NM, Noordam C, Marks HJ, Verhaak CM (2015). Parents' experiences, needs, and preferences in pediatric diabetes care: suggestions for improvement of care and the possible role of the Internet. A qualitative study. J Spec Pediatr Nurs.

[9] Haugstvedt A, Wentzel-Larsen T, Rokne B, Graue M (2011). Perceived family burden and emotional distress: similarities and differences between mothers and fathers of children with type 1 diabetes in a population-based study. Pediatr Diabetes.

[10] Nurmi MA, Stieber-Roger K (2012). Parenting children living with type 1 diabetes: a qualitative study. Diabetes Educ.

[11] Patton SR, Dolan LM, Smith LB, Thomas IH, Powers SW (2011). Pediatric parenting stress and its relation to depressive symptoms and fear of hypoglycemia in parents of young children with type 1 diabetes mellitus. J Clin Psychol Med Settings.

[12] Lowes L, Gregory JW, Lyne P (2005). Newly diagnosed childhood diabetes: a psychosocial transition for parents?. J Adv Nurs.

[13] Cousino MK, Hazen RA (2013). Parenting stress among caregivers of children with chronic illness: a systematic review. J Pediatr Psychol.

[14] Schwartz DD, Cline VD, Axelrad ME, Anderson BJ (2011). Feasibility, acceptability, and predictive validity of a psychosocial screening program for children and youth newly diagnosed with type 1 diabetes. Diabetes Care.

[15] Davis CL, Delamater AM, Shaw KH, La Greca AM, Eidson MS, Perez-Rodriguez JE, Nemery R (2001). Parenting styles, regimen adherence, and glycemic control in 4- to 10-year-old children with diabetes. J Pediatr Psychol.

[16] Tsiouli E, Alexopoulos EC, Stefanaki C, Darviri C, Chrousos GP (2013). Effects of diabetes-related family stress on glycemic control in young patients with type 1 diabetes: systematic review. Can Fam Physician.

[17] Williams LB, Laffel LM, Hood KK (2009). Diabetes-specific family conflict and psychological distress in paediatric type 1 diabetes. Diabet Med.

[18] International Diabetes Federation, International Society for Pediatric and Adolescent Diabetes (2013). Global IDF/ISPAD guideline for Diabetes in Childhood and Adolescence.

[19] Pihoker C, Forsander G, Wolfsdorf J, Klingensmith GJ (2009). The delivery of ambulatory diabetes care to children and adolescents with diabetes. Pediatr Diabetes.

[20] Ronksley PE, Sanmartin C, Campbell DJ, Weaver RG, Allan GM, McBrien KA, Tonelli M, Manns BJ, Hennessy D, Hemmelgarn BR (2014). Perceived barriers to primary care among western Canadians with chronic conditions. Health Rep.

[21] Beck JK, Logan KJ, Hamm RM, Sproat SM, Musser KM, Everhart PD, McDermott HM, Copeland KC (2004). Reimbursement for pediatric diabetes intensive case management: a model for chronic diseases?. Pediatrics.

[22] Creedy DK, Ludlow T, Beggs J, Collis D, Irvine D, Price D, Norton L, Cosgrove S, Houston K (2005). Perceptions of psychosocial support groups by parents who have a child with diabetes: a needs analysis. Collegian.

[23] Sullivan-Bolyai S, Bova C, Leung K, Trudeau A, Lee M, Gruppuso P (2010). Social support to empower parents (STEP): an intervention for parents of young children newly diagnosed with type 1 diabetes. Diabetes Educ.

[24] Doherty FM, Calam R, Sanders MR (2013). Positive parenting program (triple P) for families of adolescents with type 1 diabetes: a randomized controlled trial of self-directed teen triple P. J Pediatr Psychol.

[25] Merkel RM, Wright T (2012). Parental self-efficacy and online support among parents of children diagnosed with type 1 diabetes mellitus. Pediatr Nurs.

[26] Balkhi AM, Reid AM, McNamara JP, Geffken GR (2014). The diabetes online community: the importance of forum use in parents of children with type 1 diabetes. Pediatr Diabetes.

[27] Hawthorne K, Bennert K, Lowes L, Channon S, Robling M, Gregory JW (2011). The experiences of children and their parents in paediatric diabetes services should inform the development of communication skills for healthcare staff (the DEPICTED Study). Diabet Med.

[28] Howe CJ, Ayala J, Dumser S, Buzby M, Murphy K (2012). Parental expectations in the care of their children and adolescents with diabetes. J Pediatr Nurs.

[29] Holtslander L, Kornder N, Letourneau N, Turner H, Paterson B (2012). Finding straight answers: identifying the needs of parents and service providers of adolescents with type 1 diabetes to aid in the creation of an online support intervention. J Clin Nurs.

[30] Swift Peter G F (2009). Diabetes education in children and adolescents. Pediatr Diabetes.

[31] Boogerd EA, Noordam C, Verhaak CM (2014). The Sugarsquare study: protocol of a multicenter randomized controlled trial concerning a web-based patient portal for parents of a child with type 1 diabetes. BMC Pediatr.

[32] Nordfeldt S, Ängarne-Lindberg T, Nordwall M, Krevers B (2013). Parents of adolescents with type 1 diabetes--their views on information and communication needs and internet use. A qualitative study. PLoS One.

[33] Scharer K (2005). Internet social support for parents: the state of science. J Child Adolesc Psychiatr Nurs.

[34] Donkin L, Christensen H, Naismith SL, Neal B, Hickie IB, Glozier N (2011). A systematic review of the impact of adherence on the effectiveness of e-therapies. J Med Internet Res.

[35] Shaw RJ, Ferranti J (2011). Patient-provider internet portals--patient outcomes and use. Comput Inform Nurs.

[36] Plantin L, Daneback K (2009). Parenthood, information and support on the internet. A literature review of research on parents and professionals online. BMC Fam Pract.

[37] Van de Belt TH, Engelen LJ, Berben SA, Teerenstra S, Samsom M, Schoonhoven L (2013). Internet and social media for health-related information and communication in health care: preferences of the Dutch general population. J Med Internet Res.

[38] Grey M, Whittemore R, Jeon S, Murphy K, Faulkner MS, Delamater A, TeenCope SG (2013). Internet psycho-education programs improve outcomes in youth with type 1 diabetes. Diabetes Care.

[39] Harris MA, Hood KK, Mulvaney SA (2012). Pumpers, skypers, surfers and texters: technology to improve the management of diabetes in teenagers. Diabetes Obes Metab.

[40] Boogerd EA, Noordam C, Kremer JA, Prins JB, Verhaak CM (2014). Teaming up: feasibility of an online treatment environment for adolescents with type 1 diabetes. Pediatr Diabetes.

[41] Nicholas DB, Gutwin C, Paterson B (2013). Examining preferences for website support to parents of adolescents with diabetes. Soc Work Health Care.

[42] Eysenbach G (2005). The law of attrition. J Med Internet Res.

[43] Melville KM, Casey LM, Kavanagh DJ (2010). Dropout from Internet-based treatment for psychological disorders. Br J Clin Psychol.

[44] Murray E, Khadjesari Z, White IR, Kalaitzaki E, Godfrey C, McCambridge J, Thompson SG, Wallace P (2009). Methodological challenges in online trials. J Med Internet Res.

[45] Murray E, White IR, Varagunam M, Godfrey C, Khadjesari Z, McCambridge J (2013). Attrition revisited: adherence and retention in a web-based alcohol trial. J Med Internet Res.

[46] Verheijden MW, Jans MP, Hildebrandt VH, Hopman-Rock M (2007). Rates and determinants of repeated participation in a web-based behavior change program for healthy body weight and healthy lifestyle. J Med Internet Res.

[47] Dean SC, Harper CE, Cappuccio FP, Rink E, Dirckx C, Arnout J, Zito F, Iacoviello L, European Collaborative Group of the IMMIDIET Project (2005). The challenges of cross-national research in primary health care across Europe. Fam Pract.

[48] Mastellos N, Bliźniuk G, Czopnik D, McGilchrist M, Misiaszek A, Bródka P, Curcin V, Car J, Delaney BC, Andreasson A (2016). Feasibility and acceptability of TRANSFoRm to improve clinical trial recruitment in primary care. Fam Pract.

[49] (2016). Suikerplein.

[50] American Diabetes Association (2016). Diabetesjournals.

[51] Bowen DJ, Kreuter M, Spring B, Cofta-Woerpel L, Linnan L, Weiner D, Bakken S, Kaplan CP, Squiers L, Fabrizio C, Fernandez M (2009). How we design feasibility studies. Am J Prev Med.

[52] De BA, Vermulst A, Gerris J, Veerman J, Abidin R (2006). NOSI-R, Nijmeegse Ouderlijke Stress Index. Handleiding (NOSI-R, the Nijmegen Parenting Stress Index manual).

[53] Karlson CW, Rapoff MA (2009). Attrition in randomized controlled trials for pediatric chronic conditions. J Pediatr Psychol.

[54] McCambridge J, Kalaitzaki E, White IR, Khadjesari Z, Murray E, Linke S, Thompson SG, Godfrey C, Wallace P (2011). Impact of length or relevance of questionnaires on attrition in online trials: randomized controlled trial. J Med Internet Res.

[55] Franciscus M, Nucci A, Bradley B, Suomalainen H, Greenberg E, Laforte D, Kleemola P, Hyytinen M, Salonen M, Martin MJ, Catte D, Catteau J (2014). Recruitment and retention of participants for an international type 1 diabetes prevention trial: a coordinators' perspective. Clin Trials.

[56] Lernmark B, Johnson SB, Vehik K, Smith L, Ballard L, Baxter J, McLeod W, Roth R, Simell T (2011). Enrollment experiences in a pediatric longitudinal observational study: the Environmental Determinants of Diabetes in the Young (TEDDY) study. Contemp Clin Trials.

[57] Baxter J, Vehik K, Johnson SB, Lernmark B, Roth R, Simell T, TEDDY Study Group (2012). Differences in recruitment and early retention among ethnic minority participants in a large pediatric cohort: the TEDDY Study. Contemp Clin Trials.

[58] Aarts JW, Faber MJ, Cohlen BJ, Van OA, Nelen WL, Kremer JA (2015). Lessons learned from the implementation of an online infertility community into an IVF clinic's daily practice. Hum Fertil (Camb).

[59] Atkins S, Lewin S, Ringsberg KC, Thorson A (2011). Provider experiences of the implementation of a new tuberculosis treatment programme: a qualitative study using the normalisation process model. BMC Health Serv Res.

[60] Nijland N, van GJ, Boer H, Steehouder MF, Seydel ER (2008). Evaluation of internet-based technology for supporting self-care: problems encountered by patients and caregivers when using self-care applications. J Med Internet Res.

[61] Sharma U, Reed J, Doyle C, Bell D (2012). Challenges in evaluating telehealth through RCT-the problem of randomization. Stud Health Technol Inform.

[62] Fleuren M, Wiefferink K, Paulussen T (2004). Determinants of innovation within health care organizations: literature review and Delphi study. Int J Qual Health Care.

[63] Murray DM, Pennell M, Rhoda D, Hade EM, Paskett ED (2010). Designing studies that would address the multilayered nature of health care. J Natl Cancer Inst Monogr.

[64] West SG, Duan N, Pequegnat W, Gaist P, Des JD, Holtgrave D, Szapocznik J, Fishbein M, Rapkin B, Clatts M, Mullen PD (2008). Alternatives to the randomized controlled trial. Am J Public Health.

[65] Devine KA, Heckler CE, Katz ER, Fairclough DL, Phipps S, Sherman-Bien S, Dolgin MJ, Noll RB, Askins MA, Butler RW, Sahler OJ (2014). Evaluation of the psychometric properties of the Pediatric Parenting Stress Inventory (PPSI). Health Psychol.

[66] Schepers SA, Sint NS, Haverman L, Wensing M, Schouten van MA, Veening MA, Caron HN, Hoogerbrugge PM, Kaspers GJ, Verhaak CM, Grootenhuis MA (2017). Real-world implementation of electronic patient-reported outcomes in outpatient pediatric cancer care. Psychooncology.

