# Supplementary material for: Sugarsquare, a Web-Based Patient Portal for Parents of a Child With Type 1 Diabetes: Multicenter Randomized Controlled Feasibility Trial
Source: J Med Internet Res. 2017 Aug 22;19(8):e287. doi: 10.2196/jmir.6639 (PMC5585595; doi:10.2196/jmir.6639)

Screenshot 1: Screenshots Log-in screen

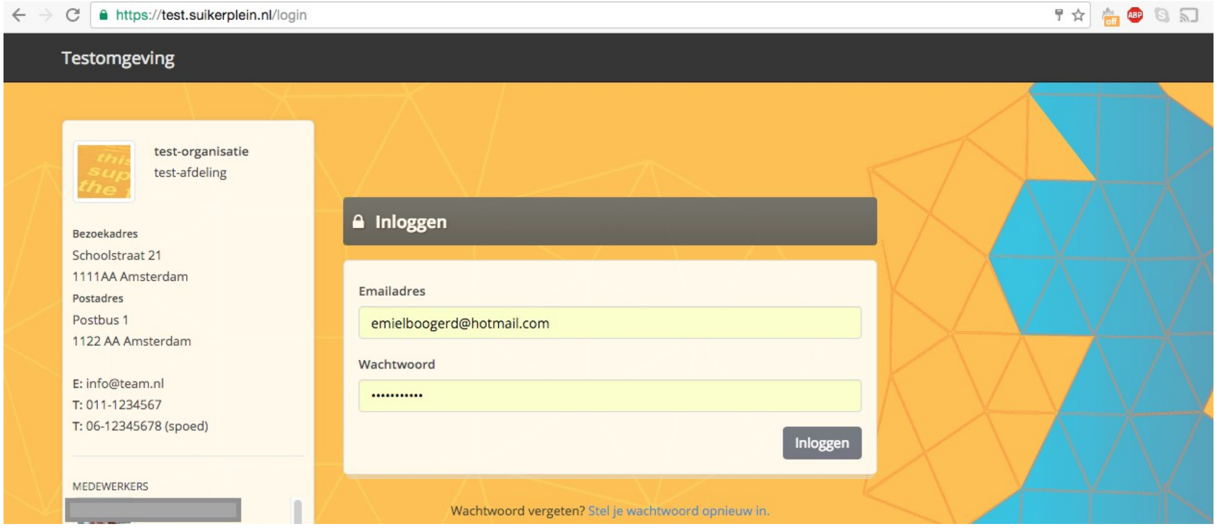

Screenshot 2: Screenshot home-screen and timeline

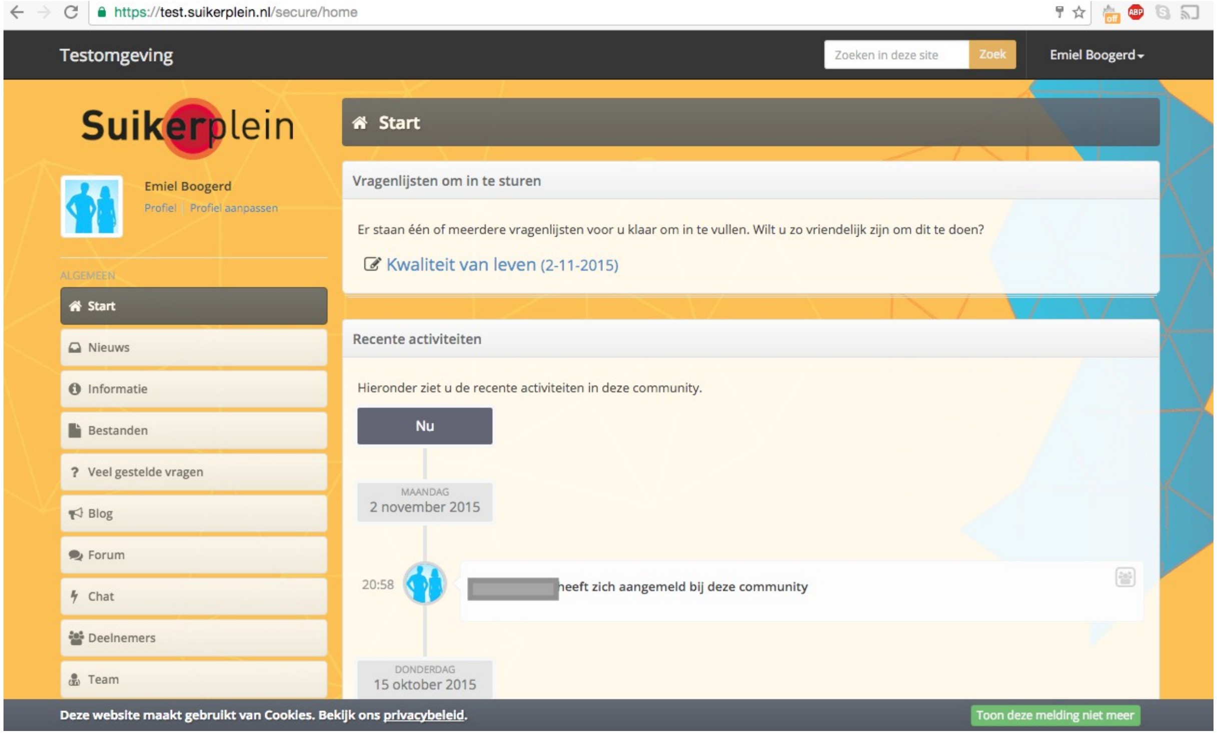

Screenshot 3: Screenshot forumdiscussion

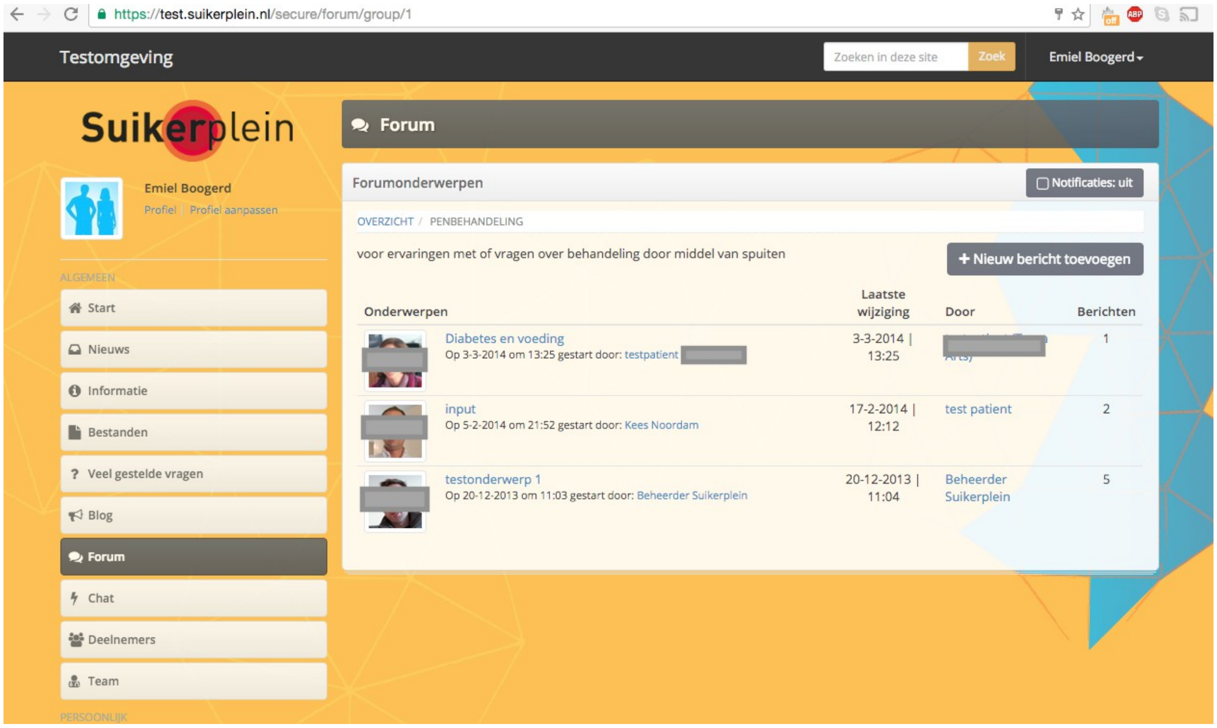

Screenshot 4: Screenshot private interaction

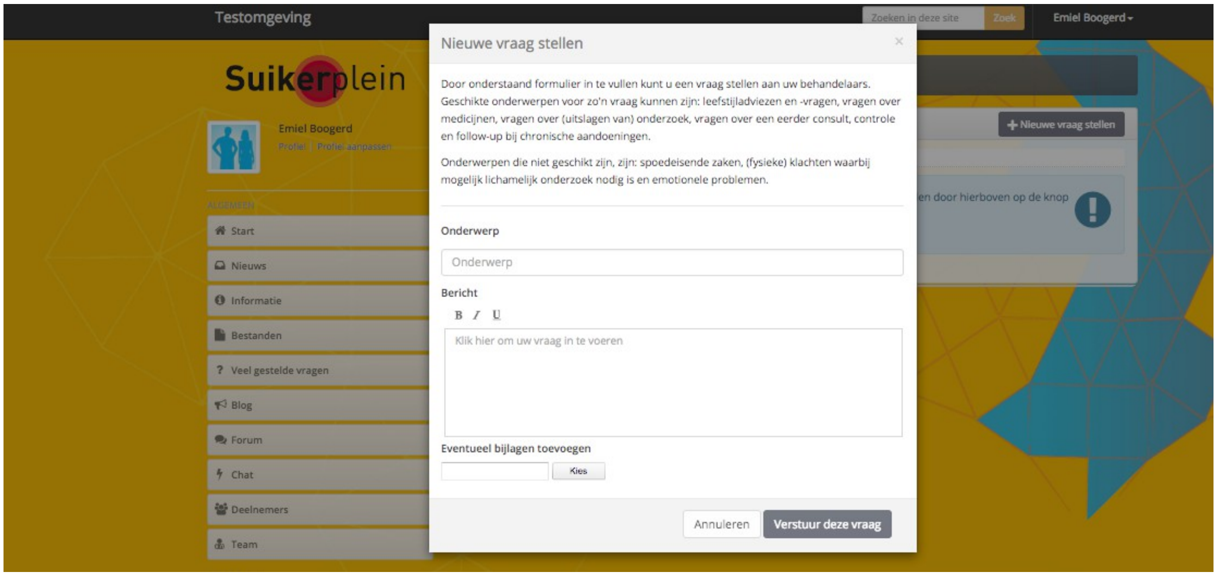

Screenshot 5: Screenshot menu

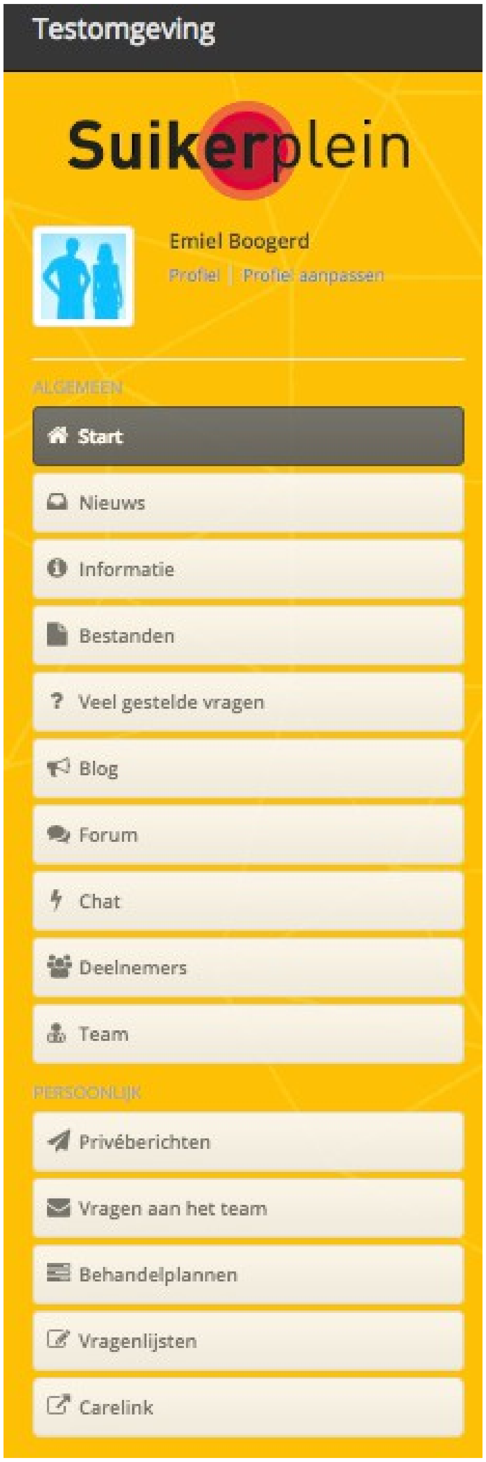

Supplement: Multimedia Appendix 1 [file jmir_v19i8e287_app1.pdf]
