# Supplementary material for: Sugarsquare, a Web-Based Patient Portal for Parents of a Child With Type 1 Diabetes: Multicenter Randomized Controlled Feasibility Trial
Source: J Med Internet Res. 2017 Aug 22;19(8):e287. doi: 10.2196/jmir.6639 (PMC5585595; doi:10.2196/jmir.6639)
Supplement: Multimedia Appendix 2 [file jmir_v19i8e287_app2.pdf]

International Guideline and Standards for Diabetes Care [18,25]	Does Sugarsquare contribute to treatment according to Guideline and Standard?	
People with diabetes should receive medical care from a physician-coordinated team.	yes	The physician-coordinated team uses the online interactive treatment environment to communicate with the parents.
Such teams may include, but are not limited to, physicians, nurse practitioners, physician's assistants, nurses, dietitians, pharmacists, and mental health professionals with expertise and a special interest in diabetes.	yes	All disciplines in the multidisciplinary teams use the online interactive treatment environment
It is essential in this collaborative and integrated team approach that individuals with diabetes assume an active role in their care.	yes	Parents are encouraged to ask questions or post comments addressing his or her child's treatment. The team can fit each other's advices to one another's.
The management plan should be formulated as a collaborative therapeutic alliance among the patient and family, the physician, and other members of the health care team.	yes	Parents are actively involved in their treatment and can discuss their child's treatment goals on the online treatment sheet.
A variety of strategies and techniques should be used to provide adequate education and development of problem-solving skills in the various aspects of diabetes management.	yes	Parents are encouraged to exchange tips about how to deal with the disease.
Implementation of the management plan requires that each aspect is understood and agreed to by the patient and the care providers and that the goals and treatment plan are reasonable.	yes	Parents can view their child's treatment goals on the online treatment sheet and discuss them online with the team.
Any plan should recognize diabetes self-management education (DSME), including introduction of new therapies and technologies, on-going diabetes support and psychosocial support as an integral component of care.	yes	The online interactive treatment environment is particularly aimed at ongoing support in-between consultations and aims at facilitating basic education and contact with the treatment team and peers. Team members can easily introduce new therapies and technologies to the parents e.g. by writing blogs.
In developing the plan, consideration should be given to the patient's age, school or work schedule and conditions, physical activity, eating patterns, social situation and cultural factors, and presence of complications of diabetes or other medical conditions.	yes	Because the online interactive treatment environment is accessible 24/7, parents can approach the team when problems occur in everyday life, instead of having to wait for a scheduled consultation.
The Diabetes Care team provides ambulatory and hospital care, including emergency access to advice, hospitalization, care visits and Quarterly HbA1c determination.	yes	The online interactive treatment environment is used as an additive to existing ambulatory and hospital care.
